# Neuronal somatic plasmalemmal permeability and dendritic beading caused by head rotational traumatic brain injury in pigs–An exploratory study

**DOI:** 10.3389/fncel.2023.1055455

**Published:** 2023-07-13

**Authors:** James P. Harris, Constance J. Mietus, Kevin D. Browne, Kathryn L. Wofford, Carolyn E. Keating, Daniel P. Brown, Brian N. Johnson, John A. Wolf, Douglas H. Smith, Akiva S. Cohen, John E. Duda, D. Kacy Cullen

**Affiliations:** ^1^Center for Brain Injury and Repair, Department of Neurosurgery, Perelman School of Medicine, University of Pennsylvania, Philadelphia, PA, United States; ^2^Center for Neurotrauma, Neurodegeneration and Restoration, Corporal Michael J. Crescenz Veterans Affairs Medical Center, Philadelphia, PA, United States; ^3^Department of Anesthesiology and Critical Care Medicine, Perelman School of Medicine, University of Pennsylvania, Philadelphia, PA, United States; ^4^Research Institute, Children’s Hospital of Philadelphia, Philadelphia, PA, United States; ^5^Department of Neurology, Perelman School of Medicine, University of Pennsylvania, Philadelphia, PA, United States; ^6^Department of Bioengineering, School of Engineering and Applied Science, University of Pennsylvania, Philadelphia, PA, United States

**Keywords:** concussion, diffuse traumatic brain injury, pig model, porcine, swine, biomechanics, neuron membrane permeability

## Abstract

Closed-head traumatic brain injury (TBI) is induced by rapid motion of the head, resulting in diffuse strain fields throughout the brain. The injury mechanism(s), loading thresholds, and neuroanatomical distribution of affected cells remain poorly understood, especially in the gyrencephalic brain. We utilized a porcine model to explore the relationships between rapid head rotational acceleration-deceleration loading and immediate alterations in plasmalemmal permeability within cerebral cortex, sub-cortical white matter, and hippocampus. To assess plasmalemmal compromise, Lucifer yellow (LY), a small cell-impermeant dye, was delivered intraventricularly and diffused throughout the parenchyma prior to injury in animals euthanized at 15-min post-injury; other animals (not receiving LY) were survived to 8-h or 7-days. Plasmalemmal permeability preferentially occurred in neuronal somata and dendrites, but rarely in white matter axons. The burden of LY^+^ neurons increased based on head rotational kinematics, specifically maximum angular velocity, and was exacerbated by repeated TBI. In the cortex, LY^+^ cells were prominent in both the medial and lateral gyri. Neuronal membrane permeability was observed within the hippocampus and entorhinal cortex, including morphological changes such as beading in dendrites. These changes correlated with reduced fiber volleys and synaptic current alterations at later timepoints in the hippocampus. Further histological observations found decreased NeuN immunoreactivity, increased mitochondrial fission, and caspase pathway activation in both LY^+^ and LY^–^ cells, suggesting the presence of multiple injury phenotypes. This exploratory study suggests relationships between plasmalemmal disruptions in neuronal somata and dendrites within cortical and hippocampal gray matter as a primary response in closed-head rotational TBI and sets the stage for future, traditional hypothesis-testing experiments.

## Introduction

Traumatic brain injury (TBI) is a major public health issue with an estimated 3.5 million incidents in the United States each year (Coronado et al., 2012). Most TBIs are closed-head, diffuse injuries that result from impact and/or inertial loading, in particular those causing rapid angular acceleration/deceleration of the head (Ommaya and Gennarelli, 1974; Adams et al., 1989; Povlishock, 1992; Smith and Meaney, 2000). Closed-head diffuse TBI has long been described as predominantly a white matter injury, characterized by the “hallmark” neuropathology of diffuse axonal injury (DAI) (Gennarelli et al., 1982; Thibault and Gennarelli, 1989; Raghupathi and Margulies, 2002; Smith et al., 2003; Browne et al., 2011; Johnson et al., 2013). However, rotational loading generally results in diffuse strain fields throughout the brain–across both gray and white matter–and the mechanisms by which neural cells sense and are pathologically affected by rapidly changing stresses remain poorly understood. The immediate physical consequences of supra-threshold loading may range from structural failure (i.e., somatic disruption or axotomy) to more subtle damage such as cytoskeletal breakdown, decoupling of organelles, and micro- or nano-tears in the plasmalemma (Pettus et al., 1994; Smith et al., 1999a; Singleton and Povlishock, 2004; Stone et al., 2004; Whalen et al., 2008; Tang-Schomer et al., 2010; Cullen et al., 2011b). In particular, evidence suggests that plasmalemma disruption, termed “mechanoporation,” can upset normal cellular function. Effects can be based on loss of membrane charge, disruption of electrokinetic transport, or osmotic imbalance (Bartoletti et al., 1989). Moreover, Ca^2+^ homeostasis may be jeopardized, directly inducing dysregulation of signaling pathways, mitochondria dysfunction, persistent dysfunction, and/or cell death (Galbraith et al., 1993; Zhang et al., 1996; LaPlaca et al., 1997, 2009; Rami et al., 1997; McIntosh et al., 1998; Villa et al., 1998; Goforth et al., 1999; Weber et al., 1999; Dumont et al., 2001; Raghupathi, 2004; Weber, 2004; Shi and Whitebone, 2006). Reported cell permeability changes are often transient (Geddes et al., 2003; Prado et al., 2005; Cullen et al., 2011b), and have been shown to present along with other types of primary neuronal and axonal injury (Singleton and Povlishock, 2004; Stone et al., 2004; Farkas et al., 2006; Kelley et al., 2006; Farkas and Povlishock, 2007) which may result in prolonged deleterious alterations in cellular function and physiology, or cell death (Singleton and Povlishock, 2004; Farkas et al., 2006; Whalen et al., 2008). Furthermore, previous work from our laboratory has demonstrated that microglial activation occurs rapidly post-injury in regions proximal to permeabilized neurons, which suggests that neuronal permeabilization may be a significant event in the initiation of neuro-inflammation following TBI (Wofford et al., 2017).

Acute alterations in cell membrane permeability are likely a direct consequence of biomechanical loading. *In vitro* studies have demonstrated that biophysical perturbations, such as high strain magnitude and rate loading, likely compromise plasma membrane integrity (Geddes et al., 2003; Prado et al., 2005; Cullen et al., 2011b). Prior *in vivo* studies using rodent models have utilized cell impermeable dyes including horse radish peroxidase (HRP), florescent dextrans, and Lucifer yellow (LY) to better understand the acute biophysical alterations in TBI (Povlishock et al., 1979; Singleton and Povlishock, 2004; Farkas et al., 2006; LaPlaca et al., 2007, 2009, 2019; Lafrenaye et al., 2012). These cell impermeant dyes were delivered to the brain parenchyma and demonstrated minimal cellular uptake prior to injury; however, brain trauma induced plasmalemmal disruptions, allowing for the intracellular influx of these dyes. Subsequent euthanasia, brain perfusion, and fixation of both tissues and intracellular dye allows identification of brain cells exhibiting compromised plasmalemma. Furthermore, use of multiple dyes permits the study of primary membrane disruption (i.e., physically induced) versus secondary disruption (i.e., biochemically induced) after the injury, which may aid in the elucidation of downstream effects (Farkas et al., 2006; Lafrenaye et al., 2012; Hernandez et al., 2019). However, rodent models of TBI may not mimic crucial neuroanatomical and neurophysiological features of human TBI pathology (Stein et al., 1993; Duhaime, 2006; Cullen et al., 2016). In contrast, pigs possess a relatively large brain mass and share key neuroanatomical similarities with the human brain, such as a gyrencephalic architecture and a 60:40 ratio of white matter to gray matter, thus enabling porcine models of TBI to more accurately represent the diffuse tissue strain regimes, and hence the biomechanical etiology, of closed-head diffuse TBI in humans (Cullen et al., 2016).

In the current study, we evaluated alterations in cellular membrane permeability in the cerebral cortex and hippocampal formation using an established large animal model of closed-head rotational TBI. Specifically, we assessed trauma-induced changes in plasmalemmal permeability via the intracellular presence of LY, a small (457Da) cell-impermeant dye, following rapid head rotational acceleration/deceleration injury in pigs. Here we report the distribution of cells exhibiting immediate alterations in plasmalemmal permeability in the cerebral cortex and hippocampus, with attention to neuroanatomical features (e.g., cortical/hippocampal sub-regions, gyri/sulci, cell layers) and as a function of head rotational kinematics (e.g., peak head rotational velocity). We also describe acute morphological alterations in neural cells exhibiting altered membrane permeability, early pathophysiological events in permeabilized cells, and early neurophysiological disruptions in hippocampal circuitry. Although closed-head diffuse TBI has classically been described as primarily manifesting as DAI throughout sub-cortical white matter, our current findings implicate additional cortical and hippocampal gray matter neuropathology based on plasmalemmal disruptions in neuronal somata and dendrites. This work contributes to a growing body of literature suggesting that trauma-induced biophysical responses and related pathophysiology are crucial drivers of neurophysiological, inflammatory, and degenerative changes. A clearer understanding of these phenomena is necessary to guide the development of effective injury prevention strategies as well as targeted neurotherapeutics following closed-head TBI.

## Materials and methods

### Animal care, anesthesia, and euthanasia

All procedures were approved by the Institutional Animal Care and Use Committee at the University of Pennsylvania and adhered to the guidelines set forth in the NIH Public Health Service Policy on Humane Care and Use of Laboratory Animals (2015). This study utilized female Yorkshire pigs (*n* = 26) with a weight range of 19–26 kg. Animals were housed indoors with unrestricted access to food and water in a facility accredited by the Association for Assessment and Accreditation of Laboratory Animal Care International. Pigs were fasted for 18–20 h prior to experiments but were provided water *ad libitum*. Anesthesia was induced *via* intramuscular injection of ketamine (20–30 mg/kg) and midazolam (0.4–0.6 mg/kg), after which they were intubated and maintained on isoflurane (1.5–2.0%, 2 L O_2_). Glycopyrrolate (0.01–0.02 mg/kg) was given during anesthesia induction to reduce tracheal secretions. Titration of anesthesia was based on pain response and the physiological parameters of SpO_2_, heart rate (HR), and respiratory rate (RR). The vitals of anesthetized animals were maintained at SpO_2_ of 97–100%, HR of 110–130 beats per minute, and RR of 12–24 breaths per minute. A surgical plane of anesthesia was maintained throughout the intraventricular injections, diffusion of LY, and injury/sham procedures. Anesthesia was also maintained between injury and euthanasia in cohorts being acutely euthanized after injury (LY injection with single injury, LY injection with same day repetitive injury, and 8-h post-injury euthanasia for electrophysiology). Anesthesia was discontinued after injury in animals assessed at the 7-day timepoint and re-induced prior to LY injection + injury and/or euthanasia. All animals received buprenorphine and acetaminophen for pain management.

**FIGURE 1:**
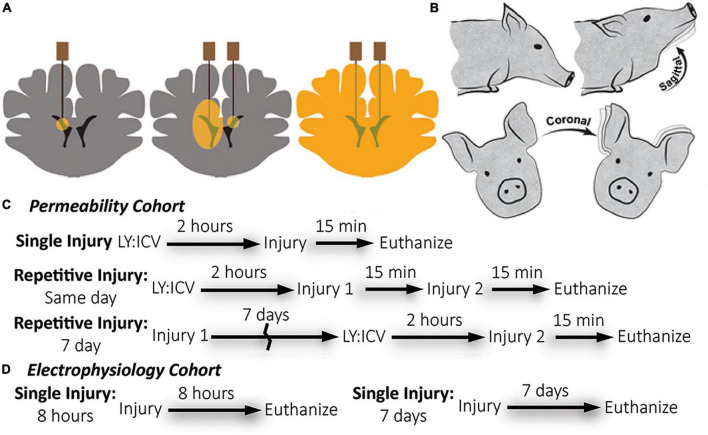
Overview of methods: Lucifer yellow (LY) delivery, rotational head injury model, and experimental timeline by cohort. **(A)** Schematic of needle placement, bi-lateral intracerebroventricular injection, and diffusion of LY throughout brain parenchyma prior to injury. **(B)** Anesthetized pigs underwent closed head rotational injury in either the coronal or sagittal plane. **(C)** The experimental timeline for the permeability cohort illustrates three different injury paradigms: single, same day repetitive, and 7 day repetitive. LY was delivered only prior to the final injury. Euthanasia occurred within 15-min of the final injury. **(D)** The experimental timeline for the electrophysiology cohort illustrates a single injury with euthanasia either at 8-h or 7-days after injury. LY was not used for the electrophysiology cohort.

Animals receiving LY injections for membrane permeability analysis were euthanized at 15-min post-injury (see Figure 1 for experimental timeline). The LY cohorts were transcardially perfused using heparinized saline (4°C, 4 L) followed by chilled 4% paraformaldehyde (4°C, 8–10 L; Sigma, P6148). The electrophysiology cohorts were transcardially perfused using a sucrose artificial cerebrospinal fluid (aCSF) (3 mM KCl, 2.5 mM NaH_2_PO_4_, 26 mM NaHCO_3_, 10 mM glucose, 1 mM MgCl_2_, 2 mM CaCl_2_, with sucrose replacing Na+ to match CSF osmolarity) chilled to 4°C. Immediately after hippocampal extraction, the remaining brain tissue was transcardially perfused with 4% paraformaldehyde fixation of the contralateral hemisphere. Prior to tissue harvest, all brains were fixed within the skull for 24 h after euthanasia in chilled paraformaldehyde to prevent fixation artifacts.

### Delivery of Lucifer yellow via intracerebroventricular (ICV) injections

Lucifer yellow (LY) is an aldehyde-fixable, small (457 Da), normally cell-impermeable dye that can be homogeneously distributed throughout the interstitial space of the brain with minimal cellular uptake. Compromise of the plasmalemma allows for cellular LY influx and subsequent visualization *via* fluorescence microscopy. As described previously (Wofford et al., 2017; Keating et al., 2020), LY (Invitrogen, L453, Carlsbad, CA, USA) was bilaterally delivered to the lateral ventricles and diffused throughout the brain parenchyma of animals before rotational/sham injury to demarcate cells permeabilized during the rotational injury. Animals were placed in a stereotactic head frame. The scalp was shaved and swabbed with betadine before a 4 cm incision was made. The scalp was reflected from the skull to visualize bregma. Two 5 mm burr hole craniectomies were made at the following stereotactic co-ordinates: 1.0 mm posterior to bregma, 6.0 mm lateral, bilaterally. LY was subsequently delivered to the ventricles in series. A small incision in the dura was made and a sterile Hamilton syringe (Reno, NV, USA) was lowered 18.0 mm from the surface of the dura to access the ventricles. Tissue was acclimated for 2 min before delivering 500 μL of LY (0.4 mg/kg in sterile saline) over a period of 10 min using an UltraMicroPump III (World Precision Instruments, Sarasota, FL, USA). The needle was slowly withdrawn at a rate of 2 mm/minute. Burr holes were sealed with bone wax. The incision site was sutured with 0–0 sutures and the area was again swabbed with betadine. Closed-head rotational/inertial injury occurred 2 h after the start of the first injection, the optimal time required to allow even LY distribution throughout the brain parenchyma. A subset of animals received a second injury either 15 min or 7 days after the initial injury in the coronal or sagittal plane. Animals undergoing repetitive injuries separated by 7 days received ICV LY injections prior to the second injury only (Figures 1A, C). Repetitive injuries separated by 15 min were chosen to represent back-to-back head impacts, which are common in contact sports, while repetitive injuries separated by 7 days were chosen to represent head impacts that occur after the initial phase of neuroinflammation and neurodegeneration has typically subsided.

### Porcine model of closed-head rotational/inertial TBI

Closed-head rotational TBI was induced under anesthesia using the HYGE pneumatic actuator, a device capable of producing pure impulsive non-impact head rotation with a controlled relationship between maximum rotational velocity/acceleration and injury severity, as previously described (Smith et al., 1997, 2000; Browne et al., 2011; Cullen et al., 2016). The well-characterized model rapidly accelerates the head and induces inertial forces representative of human TBI from falls, collisions, or blunt impacts. Angular velocity was recorded using a magneto-hydrodynamic sensor (Applied Technology Associates, Albuquerque, NM, USA) connected to a National Instruments DAQ, controlled by LabVIEW. Briefly, an animal was randomly assigned to an injury group and the animal’s head was secured to a padded bite plate and mounted to the HYGE device. In pigs, angular head acceleration in the sagittal plane (transverse to the brainstem) causes an enhancement of strain in the brainstem region compared to coronal plane accelerations (circumferential to the brainstem). Therefore, reduced levels of angular velocity/acceleration are required to elicit diffuse brain pathology, loss of consciousness, and coma for head rotation in the sagittal versus coronal plane (Meaney et al., 1995; Smith et al., 1997, 2000; Browne et al., 2011; Cullen et al., 2016). A range of kinematic loading conditions was applied for each plane in order to fully encompass clinical phenotypes ranging from “mild” to “severe” (Cullen et al., 2016). Animals were subjected to coronal plane head rotation at peak angular velocities of ∼190–300 rads/s (*n* = 7 for permeability analysis; Table 1). A separate group of animals was subjected to head rotation in the sagittal plane at peak angular velocities of ∼80–140 rad/s (*n* = 5 for permeability analysis; *n* = 4 for electrophysiology as described below). Sham animals received all other procedures absent head rotation (*n* = 3 for permeability analysis; *n* = 3 for electrophysiology as described below). In addition to the single injuries, a small group of animals received repetitive injuries (*n* = 4 total, *n* = 1 per condition) separated by 15 min or 7 days delivered in either the sagittal (15 min: 101.2 and 103.4 rad/s; 7 day: 97.2 and 100.7 rad/s) or coronal (15 min: 295.7 and 295.2 rad/s; 7 day: 282.2 and 286.2 rad/s) planes (not included in Table 1).

**TABLE 1:** Group sizes and maximum angular velocities for animals subjected to single head rotational TBI.

	Membrane permeability (LY) cohort			Electrophysiology cohort		
**Group N**	**Sham**	**Coronal**	**Sagittal**	**Sham**	**Sagittal–8 h**	**Sagittal–7 days**
1	0 rad/s	300 rad/s	139 rad/s	0 rad/s	130 rad/s	122 rad/s
2	0 rad/s	278 rad/s	130 rad/s	0 rad/s	110 rad/s	108 rad/s
3	0 rad/s	245 rad/s	114 rad/s	0 rad/s		
4		221 rad/s	93 rad/s			
5		219 rad/s	80 rad/s			
6		206 rad/s				
7		193 rad/s				

### Temporal lobectomy and hippocampal dissection for electrophysiology cohort

Anesthetized animals were transferred to a surgical suite where a craniotomy was performed to expose the entire surface of the cerebral cortex and the dura was resected. Transcardial perfusion with aCSF was initiated and subsequently the left posterior quadrant of the brain was extracted using scalpels, and the live extracted tissue was dissected on a cold block to isolate the hippocampal formation.

### Hippocampal slice acquisition and field potential recordings

The extracted hippocampus was blocked to a 1 cm section, affixed to a vibratome stage (Leica, VT1200S), and cut to a thickness of 350 μM in ice-cold (2°C) oxygenated aCSF with sucrose replacing NaCl to equal osmolarity. Slices were then incubated in oxygenated aCSF (130 mM NaCl, 3 mM KCl, 1.25 mM NaH_2_PO_4_, 26 mM NaHCO_3_, 10 mM glucose, 1 mM MgCl_2_, 2 mM CaCl_2_) at 35°C for 1 h. Field potential recordings were performed at room temperature on an interface chamber using glass electrodes (3–4 MOhm) filled with aCSF and an AxoPatch 1D amplifier in current clamp mode (Molecular Devices, CA, USA). Slices (*n* = 4–8 per animal) were continuously perfused with oxygenated aCSF. Traces were digitized and analyzed using the PClamp 10.0 software package (Molecular Devices, CA, USA). Recordings were performed in area CA1 while stimulating the Schaffer collaterals, and in the dentate while stimulating either the medial or lateral perforant path of the dorsal blade of the dentate gyrus. Care was taken to utilize the same distances between stimulating and recording electrodes, as well as between the medial and lateral perforant path. Input-output (I/O) curves were generated to examine changes in excitability in slices from injured versus sham animals. Stimulation was applied with a concentric bipolar stimulating electrode in the range of 50–500 μA. Fiber volley slopes were calculated using the linear portion of the slope. Input/output curves were initially generated using standard methodology with stimulation current as the input, but were also constructed using the measured fiber volley slope as the input compared to the excitatory post-synaptic potential (EPSP) “output.” These procedures were used for CA1 inputs from the Schaffer collaterals and for the dentate gyrus inputs from the perforant path, at both the medial and lateral portion of the dorsal blade of the gyrus.

### Statistical analysis of hippocampal field potential recordings

The experimental design for the electrophysiology analysis was modeled after a split-plot design where different injuries were administered to different animals and brain tissue from each animal was tested across a range of current inputs. Additionally, multiple brain slices were collected from each animal and considered as repeated measures. The data were analyzed using a split-plot ANOVA that incorporated two fixed factors: injury and current. To account for the repeated measures within each animal, we nested the repeated slice measurement data within each animal as previously described (Wofford et al., 2017; Wolf et al., 2017). This nesting process allows repeated measures to contribute to the effects without considering them independent units of analysis. In general, we utilized F tests to examine for an effect of injury, current, or an interaction between injury and current. If significance from the global test was detected, the data was further examined for effects at the individual group level using a Wald test.

First, mean fiber volley slope (FV slope) was assessed and characterized over ten currents, across three injury groups, and three hippocampal regions. FV slope was log transformed before being analyzed with the nested split-plot ANOVA described above, where the fixed factors were injury group and current. Secondly, mean EPSP were assessed as a function of injury group and current, using the nested split-plot ANOVA described above. EPSP data was also log transformed prior to statistical analysis. Thirdly, the relationship between FV slope input and EPSP output across all injury groups were assessed. To analyze this relationship, a mixed effects model was used where the fixed effects were current, injury group, FV slope, and the interaction between injury group and FV slope. The non-linear association of FV slope between EPSP was modeled using a cubic spline. The standard error of the mean (SEM) was calculated based on the number of animals in each experimental group. A two-sided analysis with a Type I error rate of 0.05 was applied for every test and carried out in R Studio Version 1.0.143 using R (R Core Team, 2016) version 3.2.4 and the NLME package.

### Brain tissue processing, tissue staining, and immunohistochemistry

All brains were weighed and digitally photographed from 4 aspects: right, left, ventral, dorsal. Brains were blocked in the coronal plane at 5 mm intervals, then cleared in PBS (phosphate buffered saline) and transferred to 30% sucrose until saturation. The blocks were placed in OCT, flash frozen, and stored at −80°C. Sections were cut coronally at 20–25 or 50–100 μm on a cryostat (Leica CM1860). Hematoxylin and eosin (H&E) staining was performed to evaluate general cellular changes, neuronal morphology, and pyknotic cells, as previously described (Cullen et al., 2011a). Florescent immunohistochemistry was performed with antibodies against (1) glial-fibrillary acidic protein (GFAP; rabbit IgG, 1:500; AB5804, Millipore), (2) neuronal nuclei (NeuN; mouse IgG1, 1:250; MAB377, Millipore), (3) phospho-dynamin related protein 1 (DRP1; rabbit IgG, 1:400; 3455, Cell Signaling Technologies), and (4) caspase cleaved protein (CCP; directed at caspase-derived fragment of cellular proteins; rabbit IgG, 1:1,000, 246-2, courtesy of Robert Siman). Mounted sections were washed in PBS and blocked in 2–4% non-specific horse serum with 0.3% Triton-X for 1 h at room temperature. Sections were incubated with primary antibody in blocking solution overnight at 4°C. Species-specific secondary antibodies (Life Technologies) were applied at a 1:1,000 concentration for 2 h at room temperature in blocking solution. Sections were counterstained with either Hoechst (Hoechst 33342, Life Technologies, H3570) or Fluoro-Nissl (N21479, Life Technologies). Sections were preserved with Fluoromount-G (Southern Biotech) and cover-slipped.

### Microscopy, image acquisition, and pathology scoring

Images from LY and immunohistochemically-labeled tissue sections were acquired with a confocal microscope (Nikon A1RSI Laser Scanning Confocal or Zeiss LSM 710) using 10×, 20×, 40×, or 63× objectives. Stitching of images was done in Nikon Elements AR. The anatomical location of coronal tissue sections were registered based on a magnetic resonance imaging (MRI) pig brain atlas using 3D Slicer.

#### Ordinal permeability score

A blinded scorer examined medial and lateral cortical tissue sections from each animal in the LY injury cohort. Using an ordinal scale, the scorer ranked the tissue as exhibiting 0 = absent, 1 = limited, 2 = moderate, or 3 = abundant pathology. These ordinal scores were averaged within each injury plane. A two-tailed two-way ANOVA was utilized to test for statistical differences (alpha = 0.05) with a Sidak correction for multiple comparisons. Calculations were completed in Prism v 9.5.1 (528), GraphPad Software, Inc.

#### LY pathology score across slicer atlas levels

A blinded scorer examined sections from de-identified blocks from distinct points over the rostral-caudal axis of each animal using a 10× objective. Note that the 10× objective allowed identification of fields of LY^+^ neurons within a given region, but not features of individual LY^+^ cells or processes that were better appreciated at higher magnification in subsequent analyses. A semi-quantitative approach was taken to acquire an LY pathology burden Score for each region, factoring in the presence, density, and intensity of LY+ cells. Upon identifying a region containing a patch of LY^+^ cells, the area was measured by drawing a border around the LY+ patch using the relevant tool in image viewing software. Thereafter the LY Score was multiplied by the LY Area (Score*Area) at each level to generate a score which approximates the severity and distribution of LY^+^ pathology. Finally, the Score*Area metric across each slicer level was summed to generate the Cumulative LY Score for each animal.

#### Neuron permeability and dendritic beading in the hippocampus

Images from the hippocampal region of animals with LY were collected as above and de-identified. The blinded scorer opened the stitched image in Nikon Elements AR and identified the following regions of interest (ROI): CA3, CA1, and DG. LY^+^ dendritic beads were identified and marked as present (1) or absent (0) for each animal. A statistical difference between the coronal and sagittal injuries was assessed with a two-tailed Fisher’s exact test (alpha = 0.05), which is appropriate for studies with low group numbers. Calculations were completed in Prism v 9.5.1 (528), GraphPad Software, Inc.

## Results

### TBI-induced alterations in cell membrane permeability in the cerebral cortex

**FIGURE 2:**
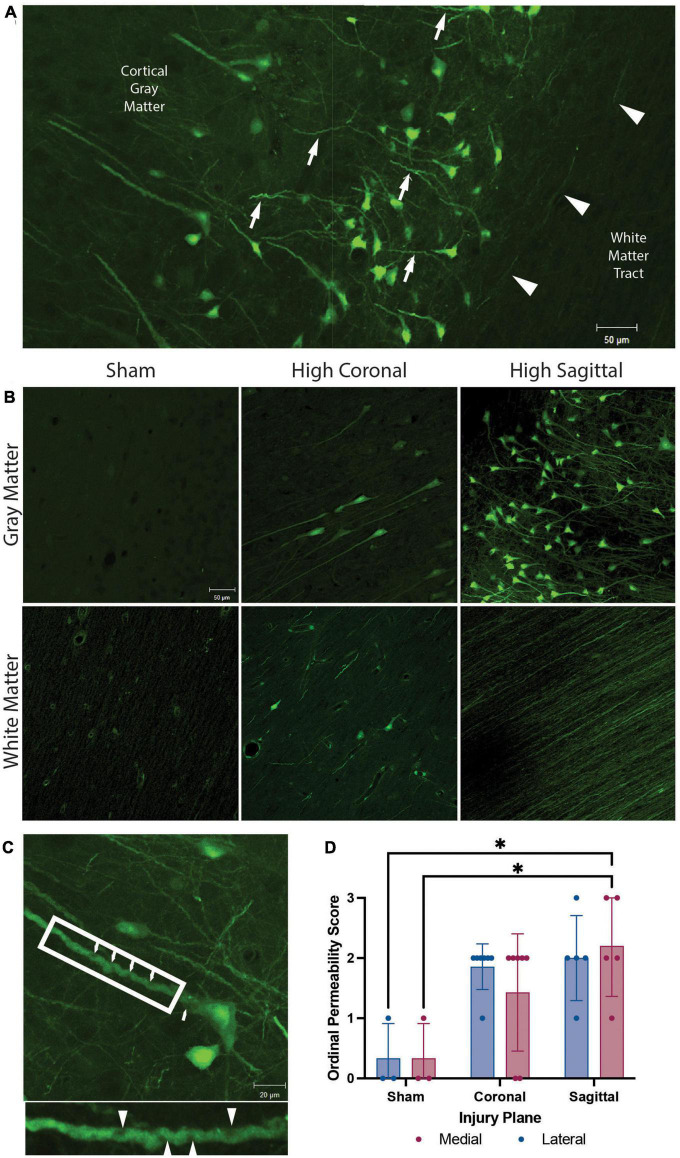
Membrane permeability and micro-structural alterations in gray matter, white matter, and gray to white matter interface. Representative images from animals with high angular velocity head rotation in the sagittal plane (130–140 rad/s), coronal plane (275–300 rad/s), and sham. **(A)** Permeability occurred within the somato-dendritic compartment, with frequent dendritic undulations (arrows), in gray matter neurons. Axonal permeability (arrowheads) was encountered in the superficial sub-cortical white matter of animals with severe sagittal injury. **(B)** Sham animals had minimal permeability. Permeability occurred more frequently in the gray matter than white matter in both coronal and sagittal injuries. White matter had limited somatic permeability and prominent axonal permeability was observed only in high angular velocity sagittal injuries (139 rad/s). **(C)** Enlargement of a neuron from (**A**) demonstrates pathological undulations and inclusions within a permeabilized neurite (arrowheads; call out box enlarged below). **(D)** Quantification of the overall LY intensity and distribution as an ordinal score from absent (score = 0) to abundant (score = 3) in medial and lateral regions of the cortex after a sham, coronal, or sagittal TBI. * denotes *p* < 0.05. Scale bars = 50 μm in **(A,B)** and 20 μm in **(C)**.

Pigs were subjected to closed-head inertial TBI at head rotational levels of 0 rad/s (sham), 80–140 rad/s in the sagittal plane, or 190–300 rad/s in the coronal plane. All animals receiving intracerebroventricular LY injections were euthanized at 15 min post-TBI (or sham conditions), and their brains processed for immunohistological and confocal microscopy examination of plasmalemmal permeability (Figure 1). We first examined the distribution and abundance of cell membrane permeability–as demonstrated by intracellular flooding with LY and subsequent sequestration–across the cerebral cortex. Sham animals exhibited few LY^+^ neurons, whereas animals subjected to head rotation in either the coronal or sagittal planes showed a marked increase in the presence of LY^+^ cells (Figure 2).

The high angular velocity sagittal injuries demonstrated the greatest extent of neuronal permeability across the cerebral cortex, often extending to subcortical gray to white matter interfaces (Figure 2A). Various imaging and post-mortem studies have demonstrated axonal injury in areas of changing tissue density, including this gray to white matter interface in human TBI (Peerless and Rewcastle, 1967; Grady et al., 1993; Povlishock, 1993; Stojanovski et al., 2019). However, while neuronal permeability was frequently seen in the perikaryon and neurites within gray matter, this phenomenon was only rarely observed within the adjacent subcortical white matter following head rotational injury (Figures 2A, B). As noted, in sham animals minimal LY uptake was observed in neurons and axons in either the gray matter or white matter (Figure 2B, left panels). However, permeabilized somata were sometimes found within white matter tracts following injury (Figure 2B, bottom center). Furthermore, axonal permeability was sporadic (Figure 2A, arrowheads) with notable axonal permeability occurring only in the most severe sagittal plane injuries (Figure 2B, bottom right). In contrast, following head rotation in either the sagittal or coronal plane, permeabilized neurons were observed in cortical gray matter across the range of head rotational velocities evaluated (Figure 2B, upper middle and upper right panels). Here, LY uptake was observed in the somato-dendritic compartment and was frequently accompanied by pathological dendritic undulations and inclusions within the gray matter (Figure 2C). Ordinal quantification of the extent of pathology in the medial and lateral regions of the cortex supported these observations, with the medial region of the cortex after a sagittal injury exhibiting a significantly higher permeability score relative to the sham group (Figure 2D).

**FIGURE 3:**
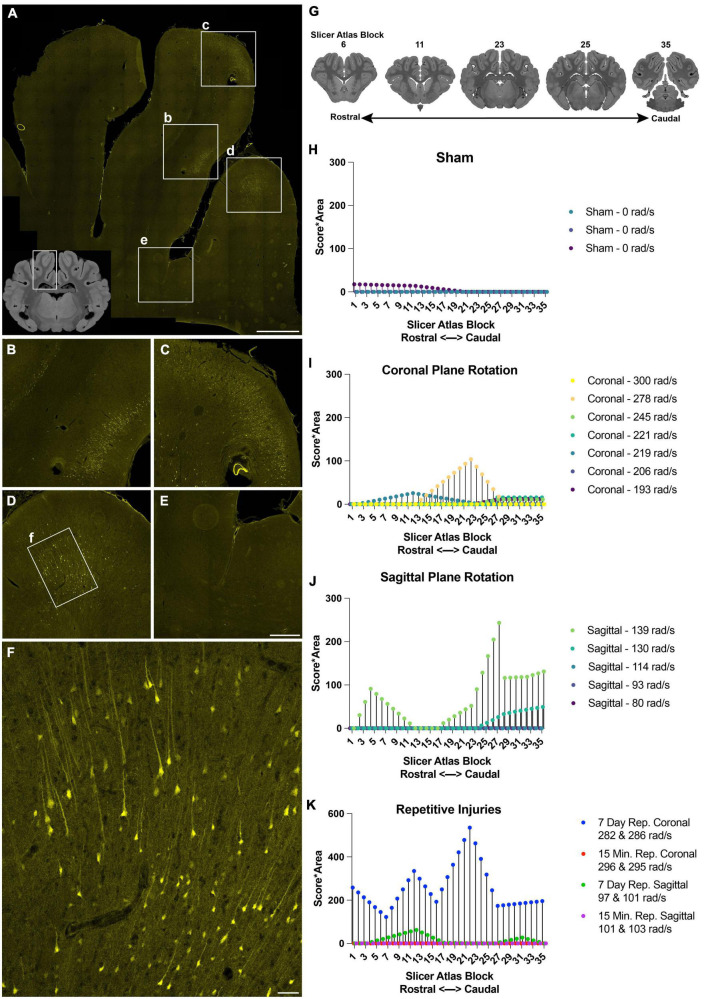
Distribution and burden of LY pathology in the cerebral cortex across injury plane and maximum angular velocity. **(A)** Hippocampal-level coronal block (representative MRI of the full block shown in inset) shows three midline gyri imaged for LY (yellow) with further magnification in boxes (b–f). There was intense LY permeability in the two midline gyri **(B–D)**, while permeability was not observed at the base of the sulci **(E)**. **(F)** Further magnification of the boxed region in **(D)** shows that LY is present throughout the cell bodies and neurites. **(G)** LY pathology was scored across the entire rostral-caudal axis for each animal, for instance in tissue sections equivalent to these illustrations from a porcine MRI brain atlas viewed in Slicer. **(H–K)** The LY pathology burden in the cortex across the rostral-caudal axis for each animal experiencing a sham, coronal plane TBI, sagittal plane TBI, or repetitive TBIs. The legend of each graph denotes the plane of head rotation and the maximum angular velocity for each animal (with two values presented for the repetitive injuries). Note the change in *y*-axis range in **(K)** relative to **(H–J)**. Scale bars = 2 mm for **(A)**, 500 μm for **(B–E)**, and 100 μm for **(F)**.

Across the cortex, LY permeability was most often seen in medial (Figures 3A–D, F) or lateral gyri (not shown), but rarely within sulci (Figure 3E). Permeability was frequently encountered within the cingulate, visual, motor, insular, and prepiriform cortices. Limited permeability was observed in the parahippocampal and piriform cortices. Across various anatomical levels of the cortex, the distribution of permeabilized cells exhibited a multi-focal or “skip phenomenon”: patches of LY^+^ cells were present within some gyri but absent in others, including adjacent gyri. LY was sequestered throughout cell bodies and neurites (Figure 3F), consistent with previous reports using this fluorophore (LaPlaca et al., 2009; Simon et al., 2009; Wofford et al., 2017; Keating et al., 2020). In general, we observed head rotation in the sagittal plane resulted in a greater incidence of permeability than rotation in the coronal plane at the angular velocity levels tested. Moreover, repetitive injuries resulted in more permeabilized neurons than single injuries.

To further describe the relationship between injury kinematics–specifically maximum angular velocity–and LY pathology across injury planes and brain location, we quantified LY pathology across the rostral-caudal axis for each animal. The extent of LY pathology was identified by a blinded scorer observing the tissue through a 10× objective, allowing visualization of patches of LY^+^ neurons, but not allowing detection of individual LY^+^ neurons or dendritic pathologies. Across the cortex, we observed that sham animals exhibited minimal LY pathology (Figure 3H). Following an injury in the coronal plane, we observed pathology distribution that was variable across the rostral-caudal axis for each animal (Figure 3I). Animals experiencing higher velocity sagittal injuries exhibited more pathology, most prevalently in the caudal aspect of the cortex (Figure 3J). Repetitive injuries also increased the extent and distribution of LY pathology in the cortex (Figure 3K). In general, single coronal or sagittal plane injuries at the lower angular velocity levels generated modest cortical pathology. Surprisingly, we observed that repetitive injuries in the coronal plane separated by 15 min (injured with similar injury kinematics) exhibited low levels of LY pathology in the cortex, similar to the trend of a single coronal injury. However, repetitive injuries in the coronal plane separated by 7 days exhibited amplified pathology in the cortex relative to animals injured with a single TBI at similar kinematics (Figure 3K). Furthermore, repetitive injuries in the sagittal plane separated by 15 min also exhibited minimal LY pathology, similar to animals injured with a single TBI with comparable kinematics. In contrast, repetitive injuries in the sagittal plane separated by 7 days exhibited marginally increased LY pathology in the cortex relative to animals experiencing a single injury with similar injury kinematics (Figure 3K).

**FIGURE 4:**
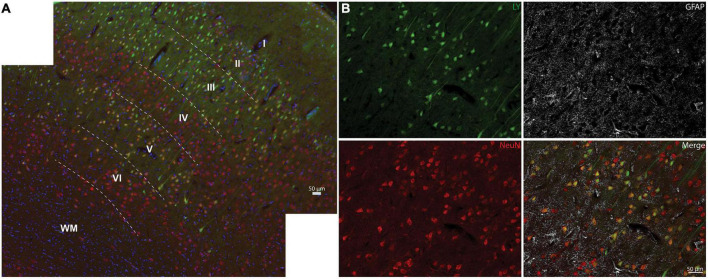
Neocortical distribution of LY^+^ neurons. **(A)** Representative images from a severe sagittal injury (130–140 rad/s) labeled with LY (green), NeuN (red), and Hoechst (blue). Cells with LY uptake were predominantly in layers II, III, and V, with sporadic permeability in layer VI. **(B)** Magnification of Layer V from **(A)** demonstrates that permeabilized cells (LY+) were neuronal (NeuN+), not astrocytic (GFAP, white). Dotted lines denote cortical layers, superficial (I) through deep (VI), and white matter (WM). Scale bars = 50 μm.

We next examined the distribution of permeabilized cells within the layers of the neocortex (Figure 4). The cortical layers affected by LY uptake depended on the overall burden of LY permeability. In areas with limited permeability, cortical layers II and III were predominantly affected, showing low to high intensity of LY uptake. As the loading severity increased based on rotational levels in either the sagittal or coronal planes, the density and distribution of permeabilized cells also increased. For the low angular velocity coronal injuries, LY permeability occurred most frequently in cortical layers II and III, but in some cases isolated permeability was observed in layer V. In cortical layer V, permeability appeared to be selective to large pyramidal cells. Higher angular velocity coronal injuries had a wider array of LY^+^ cells in layers II, III, and V. All of the angular velocity levels evaluated in the sagittal plane demonstrated some degree of permeability in layers II, III, V, and sometimes VI (Figure 4A). Layers I and IV were largely devoid of LY^+^ neuronal permeability; this was the case across the full range of loading severity and planes examined. Given the selective distribution of permeabilized cells, we next confirmed the phenotype of afflicted cells and observed that permeabilized cells were exclusively neurons (Figure 4B), consistent with our previous reports (Wofford et al., 2017; Keating et al., 2020).

**FIGURE 5:**
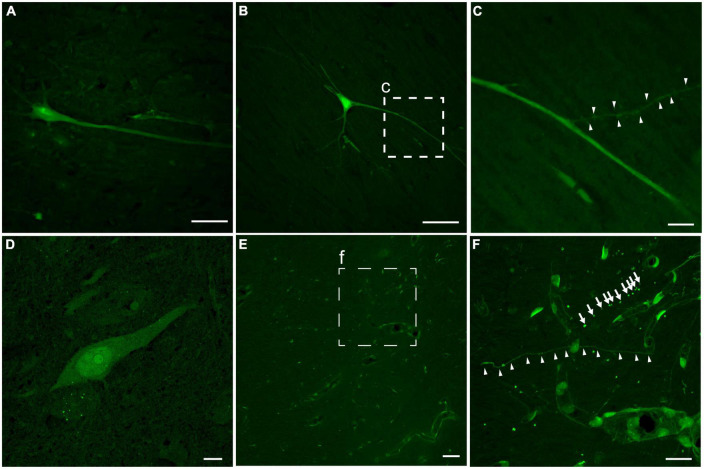
Intracellular distribution and intensity of neuronal LY. Example images of neurons from an animal with repetitive head rotation in the coronal plane at 282 and 286 rad/s separated by 7 days. **(A)** Contiguous LY within the neuronal soma and apical dendrite demonstrating decreasing LY intensity with increasing distance from the soma. **(B)** Neurons demonstrated LY uptake spanning significant distances throughout the dendritic tree. **(C)** Enlargement of callout box in **(B)** demonstrating that LY uptake was observed in dendritic spines (arrowheads). **(D)** Neuronal soma demonstrating increasing LY intensity between the cytoplasm, nucleus, and nucleolus, respectively. **(E)** In cortical areas with LY uptake grossly limited to the vasculature, **(F)** closer inspection [enlargement of callout box in **(E)**] revealed LY uptake within both morphologically normally appearing neurites (arrowheads) and beaded neurites (arrows). Scale bars = 50 μm for **(A,B,E)**; 10 μm for **(C,D,F)**.

In addition to the regional burden of LY pathology, the distribution of intracellular LY labeling also varied, but was generally observed in multiple intracellular compartments of neurons following both single and repetitive injuries (Figure 5). A subset of neurons demonstrated isolation of LY to the somatic region. However, other neurons demonstrated contiguous LY uptake from the soma to the neurites, with decreasing intensity of LY along the neurite as the distance from the soma increased (Figures 5A–C). In some cases, LY spanned a significant distance down the dendritic tree, even flooding dendritic spines (Figure 5C). Increasing intensity of LY from the cytoplasm to the nucleus, to the nucleolus, was frequently observed as well (Figure 5D). Dendritic flooding with LY also revealed beading within these neurites in the cortex (Figures 5E, F), and this dendritic beading was more frequent in animals receiving repetitive injuries.

### Acute pathologies co-occurring with plasmalemmal permeability in the cerebral cortex

#### Cells with LY permeability show changes in NeuN antigenicity

**FIGURE 6:**
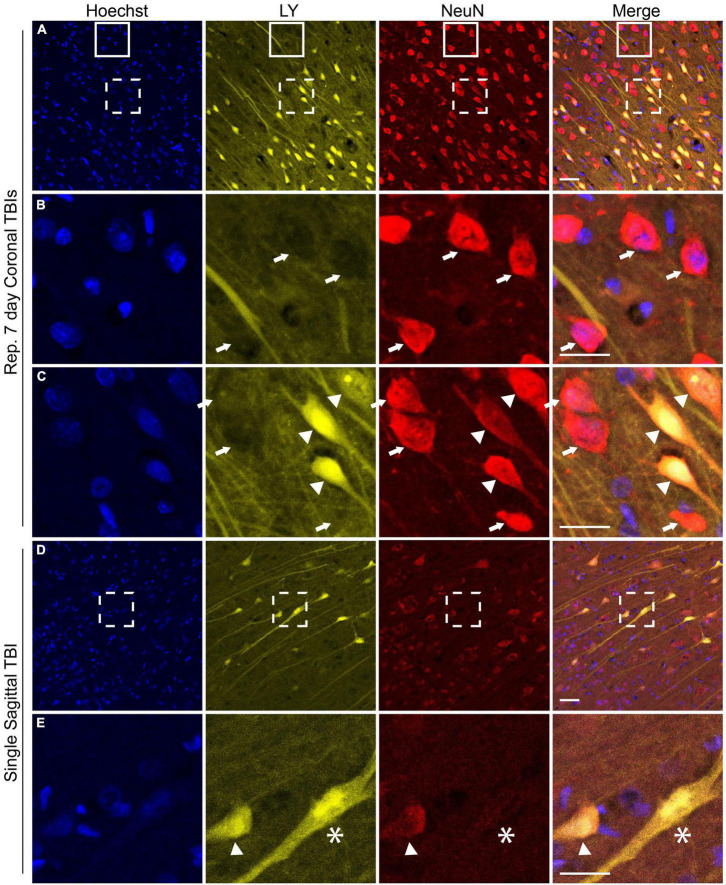
NeuN expression is altered in the cerebral cortex following both single and repetitive closed-head rotational injury. Representative images from (**A–C**) animals receiving repetitive high velocity coronal injury (282 and 286 rad/s, 7-day separation between injuries) or **(D,E)** a single high velocity sagittal injury (130–140 rad/s), immunolabeled for Hoechst (blue), LY (yellow), NeuN (red), and overlay. **(A)** NeuN expression was variable in animals receiving repetitive injuries. **(B)** Magnification of solid box in **(A)** revealed LY^–^ neurons with robust somatic NeuN expression (arrows). **(C)** Magnification of dashed box in **(A)** revealed LY^–^ cells with robust somatic NeuN expression (arrows) and LY^+^ cells with variably diminished somatic NeuN expression and dispersion of NeuN labeling to the hillock/neurites (arrowheads). **(D)** Animals receiving a high angular velocity single sagittal injury demonstrated variable loss of NeuN expression in cortical cells. **(E)** Magnification of the boxed region in **(D)** demonstrated that LY^+^ cells have diminished (arrowhead) or absent (asterisk) NeuN expression. Scale bars = 50 μm for **(A)** and **(D)**, 25 μm for **(B,C,E)**.

NeuN is exclusively expressed in neuronal tissues (Mullen et al., 1992) and is localized in the nuclei and perinuclear cytoplasm (Gusel’nikova and Korzhevskiy, 2015). Loss of NeuN antigenicity, which may arise from alteration in protein phosphorylation (Lind et al., 2005), has been associated with neuronal injury and delayed neurodegeneration (Igarashi et al., 2001; Davoli et al., 2002; McPhail et al., 2004; Ünal-Çevik et al., 2004; Tippett et al., 2007; Kirik et al., 2009; Korzhevskii et al., 2009). We recently reported changes in NeuN expression and/or distribution that co-occurred with changes in neuronal plasmalemmal permeability in sub-cortical regions after repetitive closed-head rotational injury in pigs (Keating et al., 2020). To determine if similar findings were present within the cerebral cortex after either single or repetitive injury, we performed NeuN labeling and analysis. In animals receiving repetitive injuries, we observed a similar variable pattern of NeuN labeling in the cortex as was previously observed in sub-cortical regions (Figures 6A–C). Neurons without LY uptake had typical nuclear and peri-nuclear labeling (Figures 6A–C, arrows). However, permeabilized neurons demonstrated variable NeuN labeling intensity as well as dispersion of NeuN immunoreactivity to the hillock/neurites (Figures 6A, C arrowheads). In our cohort of single injury animals, we observed that animals with extensive cortical LY permeability (following high rotational velocity injury) also demonstrated varied NeuN expression (Figures 6D, E). In these permeabilized cortical cells, NeuN expression was either retained, diminished (Figure 6E arrowhead), or absent (Figure 6E asterisk).

#### Mitochondrial fission

**FIGURE 7:**
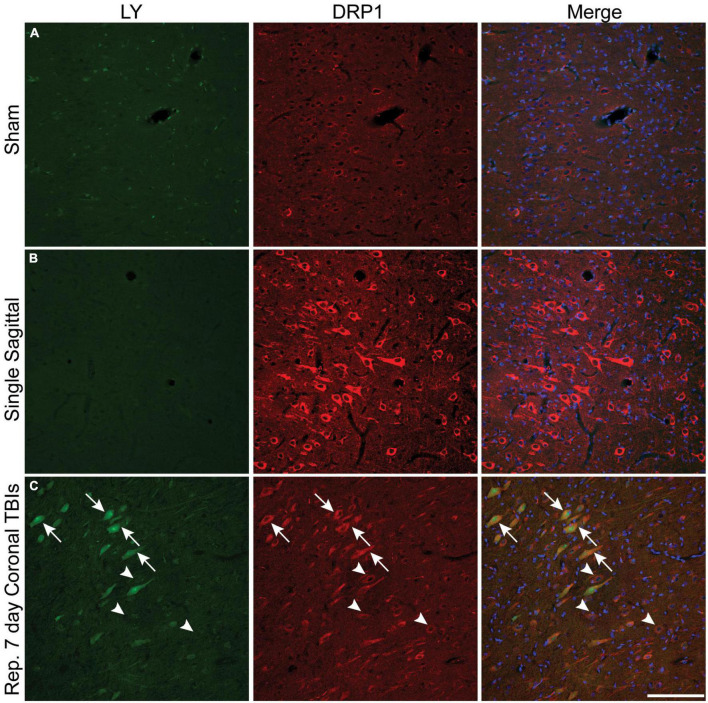
Metabolic stress is increased after closed-head rotational injury and is observed within permeabilized cells. Representative images from **(A)** sham, **(B)** single high sagittal injury (130–140 rad/s), and **(C)** repetitive coronal injury (282 and 286 rad/s, injuries separated by 7 days) animals labeled for DAPI (blue), DRP1 (red), and LY (green). **(A)** Baseline DRP1 expression and absence of permeabilized cells is demonstrated in a sham animal. **(B)** Cortical areas with minimal permeability demonstrated increased DRP1 expression after single injury. **(C)** Increased DRP1 expression was consistently encountered within permeabilized cells (arrows), but was also encountered in non-permeabilized cells (arrowheads) within the cortex of animals receiving repetitive injuries. Scale bar = 50 μm.

Plasmalemmal permeability likely disrupts intra-neuronal ion gradients, potentially resulting in significant metabolic stress as homeostatic ion concentration gradients are reestablished. We utilized phospho-dynamin related protein 1 (DRP1), an essential GTPase for mitochondrial fission (Fonseca et al., 2019), as our surrogate marker for increased cellular energetic demand to evaluate if metabolic stress is more prominent within permeabilized cells. Faint DRP1 and minimal LY labeling was observed in sham animals (Figure 7A). After a single rotational injury, we identified regions exhibiting a clear increase in DRP1 expression in the soma, with some cells demonstrating extension of DRP1 labeling into the apical dendrite. Although the majority of cells with increased DRP1 labeling were not LY^+^ (Figure 7B), permeabilized cells frequently had increased DRP1 labeling. Interestingly, the prevalence of permeabilized LY^+^ cells with increased DRP1 labeling increased with repetitive injury within the cortex (Figure 7C, arrows). As in single injury, repetitive injury also demonstrated increased DRP1 labeling in LY^–^ cells (Figure 7C, arrowheads).

#### Caspase pathway activation

**FIGURE 8:**
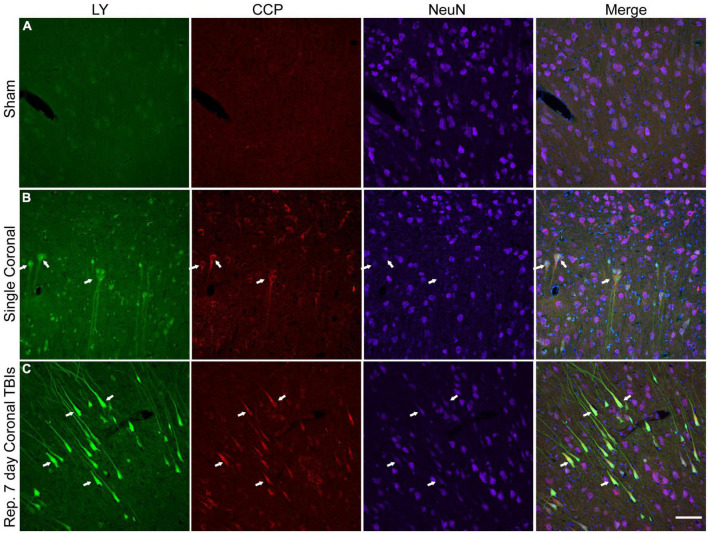
Caspase pathway activation, neuronal permeability, and altered NeuN expression in the cortex of animals receiving either single or repetitive injury. Representative images from **(A)** sham, **(B)** single high coronal injury (280–300 rad/s), or **(C)** repetitive high coronal injury (282 and 286 rad/s, injuries separated by 7 days) animals were labeled with LY (green), caspase-cleaved protein (CCP, red), NeuN (purple), and Hoechst (blue). **(A)** Sham animals had minimal cortical LY or CCP labeling. **(B)** Faintly LY^+^ cells co-localized with CCP (arrows) and also demonstrated a reduction or loss (right arrow) of NeuN labeling in animals receiving a single injury. **(C)** Areas of significant permeability show frequent co-localization of LY^+^ cells with CCP (arrows). LY^+^/CCP^+^ cells exhibited diminished NeuN labeling. Scale bar = 100 μm.

We explored the potential relationship between plasmalemmal permeability and early cell death pathways. Here, we labeled for caspase-cleaved protein (CCP), a marker that has been previously shown to align well with activation of the apoptotic enzyme caspase-3 (Chen et al., 2005). Minimal CCP or LY labeling was observed in the cortex of sham animals (Figure 8A). Single high-level injury in either the sagittal or coronal plane resulted in increased neuronal CCP labeling (Figure 8B). Likewise, increased neuronal CCP labeling was observed in the cortices of animals receiving repetitive injuries and was more prominent than the CCP labeling observed in single injuries (Figure 8C). The majority of LY^+^ soma and neurites (including neurons with only faint LY uptake) co-localized with CCP, although CCP labeling was not exclusive to permeabilized neurons (Figures 8B, C). Notably, NeuN immunoreactivity was diminished (Figures 8B, C) or lost (Figure 8B, right arrow) in CCP^+^/LY^+^ cells.

### LY^+^ neuronal morphology and intracellular distribution in the hippocampus

**FIGURE 9:**
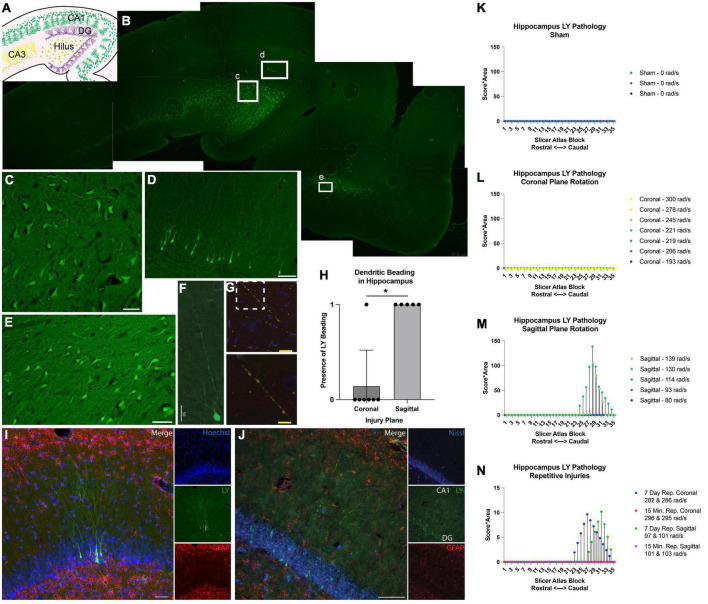
Hippocampal permeability and dendritic beading in single and repetitive injury. **(A)** A schematic of hippocampal anatomy. **(B)** Stitched image of the hippocampus from an animal subjected to a single high rotational velocity injury in the sagittal plane (130–140 rad/s). Magnification of callout boxes from **(B)** reveal several LY^+^ (green) permeabilized cells in the **(C)** hilus, **(D)** dentate gyrus, and the **(E)** entorhinal cortex. **(F,G)** Permeabilized dentate granule cells demonstrated distinct phenotypes with either contiguous dendritic uptake of LY (high sagittal injury, 130–140 rad/s), or prominent dendritic beading (high sagittal injury, 130–140 rad/s). Image was labeled with neurofilament (red), LY (yellow/green), and Nissl (blue). Enlargement of call out box in **(G)** demonstrated a “string-of-pearls” morphology. **(H)** Quantification of the presence of LY beading in the hippocampus after coronal or sagittal plane rotational injuries (all rotational levels combined for each plane). **(I)** A representative image of the dentate granular layer from a single high rotational velocity sagittal injury (130–140 rad/s) demonstrated LY (green) distribution that is uniform from the soma and extended deeply and contiguously into the dendrites within permeabilized dentate granular cells (Hoechst; blue). Astrocytic permeability was not observed (GFAP; red). **(J)** Repetitive coronal injury (282 and 286 rad/s, separated by 7 days) demonstrated LY uptake (green) in the somato-dendritic compartment, as well as prominent dendritic beading of dentate granular layer neurons (Nissl; blue). Astrocytic permeability was not observed (GFAP; red). To illustrate the effect of injury plane and maximum angular velocity on pathology throughout the hippocampus, the LY pathology burden was plotted against levels from a porcine MRI atlas (lower atlas levels represent rostral brain regions while higher atlas levels represent caudal regions). **(K–N)** LY pathology burden in the hippocampus of individual animal replicates experiencing **(K)** sham procedure, **(L)** coronal plane rotation, **(M)** sagittal plane rotation, or **(N)** repetitive injuries. The legend of each graph denotes the plane of head rotation and the maximum angular velocity for each animal (with two values presented for the repetitive injuries). Note the change in *y*-axis range in **(N)** relative to **(K–M)**. Data in **(H)** is presented as mean ± SD where * denote *p* < 0.05. Scale bars = 50 μm **(C–F,I,J)**; 25 μm **(G)** and 10 μm (**G**, inset).

Memory and attention deficits are commonly seen after human TBI, likely due to damage to the hippocampal formation and/or cortical structures providing inputs to the hippocampus. Therefore, we next examined the hippocampus for permeabilized cells and any structural alterations. In injured animals, LY^+^ neurons were found in several different hippocampal regions (Figures 9A, B), including the hilus (Figure 9C), dentate gyrus (Figure 9D), CA1, CA3, and also in the entorhinal cortex (Figure 9E). Similar to the cortex, LY uptake was exclusively neuronal and was frequently observed within the somato-dendritic compartment of hippocampal neurons (Figures 9C–F). Interestingly, LY uptake extended particularly far into the dendrites of dentate granule cells (Figures 9F, I) and often displayed LY^+^ varicosities (beading) along the dendritic processes (Figure 9G). Several distinct beading phenotypes were observed in the dentate gyrus. In some cases, the LY distribution was more uniform between the cell bodies and dendrites (Figures 9F, I). In others, cell bodies were filled with LY, and dendrites had a “string-of-pearls” appearance (Figures 9G, J).

The amount of hippocampal dendritic beading observed depended on the severity and plane of injury. Dendritic beading was not observed in any of the sham animals or in any of the animals subjected to single coronal plane head rotation at 190–245 rad/sec, but dendritic beading was observed in one animal subjected to single coronal plane rotation above 275 rad/sec. In this case, hippocampal neuronal permeability was limited to a few scattered neuronal soma following high angular velocity coronal plane head rotation. However, hippocampal dendritic beading was observed in all animals subjected to head rotation in the sagittal plane, regardless of the rotational velocity level (Figure 9H).

Given the contrast in hippocampal permeability findings between coronal and sagittal injuries, we next examined the morphologic features of LY^+^ neurons in animals receiving repetitive injuries in either the coronal or sagittal plane. All animals who received a second injury, regardless of injury plane or timing of secondary insult (same day or 7 days), demonstrated neuronal somato-dendritic permeability within the hippocampus. Intriguingly, whereas dendritic permeability was scarce in single high coronal injuries, repetitive high angular velocity coronal injuries demonstrated a higher prevalence of dendritic beading and permeability (Figure 9J).

To further support these observations, we quantified the amount of LY pathology across the rostral-caudal axis to map the effect of injury plane and velocity on hippocampal pathology. As in the cortex, a blinded scorer observed the tissue through a 10× objective, allowing detection of patches of LY^+^ neurons, but not allowing detection of individual LY^+^ neurons or fine dendritic pathologies. We observed no widespread patches of LY+ neurons across any rostral-caudal block after a sham, coronal (at any angular velocity), or sagittal injury of 114 rad/s or below (Figures 9K–M). For animals experiencing sagittal injuries at 130 rad/s or above, we observed a drastic increase in the extent of hippocampal pathology. Repetitive injures also affected hippocampal pathological burden. As noted, single coronal injuries at 278–300 rad/s generated no widespread patches of LY+ cells, but repetitive injuries in the angular velocity range separated by 7 days, but not 15 min, generated readily observable pathology (Figure 9N). Furthermore, whereas single sagittal injuries in the range of 93–114 rad/s did not generate hippocampal pathology, repetitive injuries in this range separated by 7 days, but not 15 min, generated readily detectable pathology (Figure 9N).

### Relationship between cumulative LY^+^ burden and head rotational kinematics

**FIGURE 10:**
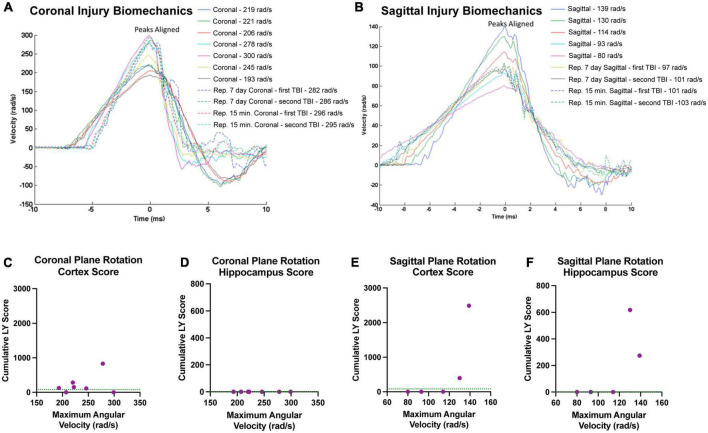
Relationships between injury biomechanics and cumulative LY pathological burden. The angular velocity curve for the head rotation experienced by each animal in the **(A)** coronal or **(B)** sagittal plane is plotted and overlayed atop one another. The maximum angular velocities (the peak of each graph) were aligned for ease of comparison. Cumulative LY pathology burden (based on measures of patches of LY+ cells) in the **(C,E)** cortex or **(D,F)** hippocampus was plotted against maximum angular velocity for each animal after **(C,D)** coronal plane rotation or **(E,F)** sagittal plane rotation with each point representing an individual animal. Sham animals experienced no head rotation (0 rad/s) and **(C,E)** exhibited minimal pathology in the cortex and **(D,F)** no pathology in the hippocampus. The average pathology score for the sham animals in each region is denoted as a green horizontal dashed line in the graphs.

Our findings in the cortex and hippocampus suggest an association between the maximum angular velocity and pathological outcome. Therefore, we plotted each animals’ individual injury angular velocity trace over time in the coronal (Figure 10A) and sagittal (Figure 10B) planes. The maximum angular velocities (the peak of each velocity trace) were aligned for each animal for ease of comparison. The injury kinematics spanned a range of velocities, allowing us to investigate trends in the relationship between maximum angular velocity and LY pathology across injury planes. Accordingly, we plotted each animal’s maximum angular velocity against their cumulative LY burden, which was the extent of LY pathology summed across brain sections (i.e., per-animal rostral-caudal summation of Score*Area presented in Figure 3 for cortex and Figure 9 for hippocampus). Within the cortex, sham animals experienced no rotational injury (0 rad/s) and exhibited either no pathology (2/3 animals in sham cohort) or minimal pathology (1/3 animals in sham cohort), as denoted by the horizontal dashed line in Figures 10C, E. Increasing maximum angular velocity in a coronal rotational injury generally correlated with an increase in the cumulative LY score in the cortex (Figure 10C). Increasing maximum angular velocity in a sagittal rotational injury generated increasing LY cortical pathology that mimicked an exponential trend, where injuries below 120 rad/s caused negligible patches of LY pathology while injuries above 120 rad/s induced abundant patches of LY pathology (Figure 10E). Within the hippocampus, following sham procedure or any of the angular velocities in the coronal plane, we did not observe any major patches of LY+ cells (Figure 10D). However, the pathological burden in hippocampus following head rotation in the sagittal plane generally increased with higher angular velocities. Similar to the trend in the cortex, sagittal injuries below 120 rad/s were associated with minimal patches of LY pathology while injuries above 120 rad/s induced widely observable patches of LY pathology (Figure 10F).

### Hippocampal electrophysiology after sagittal TBI

**FIGURE 11:**
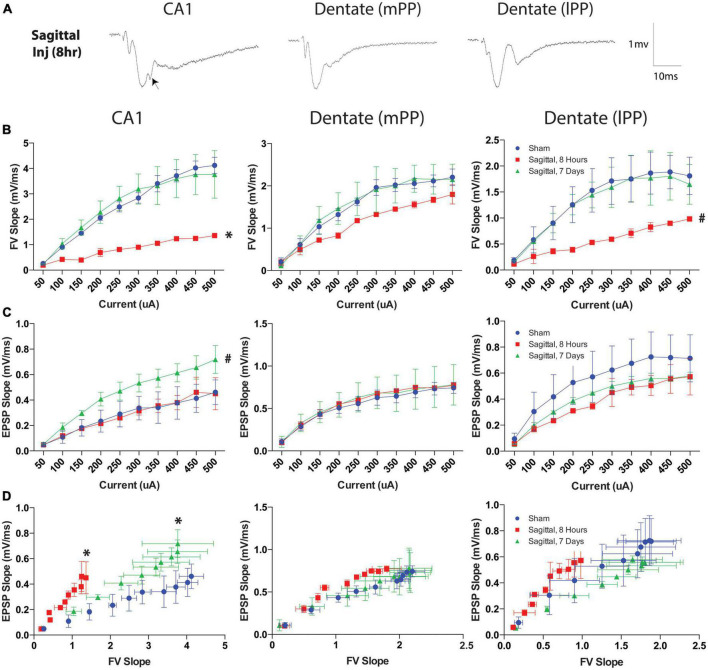
Current and fiber volley inputs affect EPSP after severe TBI. **(A)** Representative examples of responses to stimuli in the Schaffer collaterals (CA1) or medial (mPP) and lateral (lPP) perforant path (DG) in hippocampal slices after rotational brain injury. Each waveform is the average of ten traces at the half-maximal stimulation. Note the disruption in the EPSP post-injury in area CA1 when stimulating the Schaffer collaterals (arrow). **(B)** Fiber volley (FV) output was transiently reduced in the CA1 8-h after injury but returned to sham levels by 7-days after injury. This trend was less pronounced in the dentate lateral pathway (*p* = 0.077) and not present in the medial pathway. **(C)** Downstream EPSP output was measured over a range of currents after TBI. The Schaffer collaterals pathway exhibited a slight increase in EPSP 7-days after injury (*p* = 0.077). EPSP measurements in the lateral and medial perforant pathways were not significantly different from sham injured animals at either acute or subacute time points. **(D)** To evaluate the effect of fiber volley slope on EPSP output after injury, the data were plotted as a function of FV slope at each current within each region. Eight hours after injury, a given fiber volley input generated an enhanced EPSP output in the Schaffer collaterals pathway, which was preserved 7-days after injury. This trend was not observed in either the lateral or medial perforant pathways of the dentate. Data presented at mean ± SEM and where * denotes *p* < 0.05 and # denotes *p* < 0.10 relative to sham.

Since sagittal injuries produced the greatest hippocampal permeability, we examined a separate cohort of animals injured in this plane to characterize neurophysiological changes utilizing a previously characterized hippocampal slice recording paradigm (Wolf et al., 2017). Electrophysiological recordings from three regions of the hippocampus were quantified for mean fiber volley slope (FV slope) and excitatory postsynaptic potential (EPSP) following current stimulation ranging from 50 to 500 μA. Representative examples of responses to stimuli are presented in Figure 11A. We observed notable suppression of presynaptic FV slope at 8-h post-injury in the CA1 region (Schaffer collaterals) (*p* = 0.011). Interestingly, this effect was not present at 7-days post-injury at the rotational levels investigated, suggesting a transient phenomenon (Figure 11B). The medial perforant pathway (mPP) of the dentate did not exhibit the same shift in FV slope at either the acute 8-h or the subacute 7-day time points (Figure 11B). FV slope in the lateral perforant pathway (lPP) of the dentate showed a non-significant trend toward a similar transient decrease (*p* = 0.077; Figure 11B). Conventional input-output curves also revealed a non-significant trend toward increased excitatory postsynaptic potential (EPSP) slope in 7-day sagittal injury animals in Schaffer collaterals pathway (*p* = 0.077), however no differences were observed in either the mPP or lPP (Figure 11C). We next analyzed the relationship between the axonal input (FV) and the synaptic current by plotting the EPSP slope against the fiber volleys at each current input. This analysis revealed that a given fiber volley input led to a greater EPSP output in area CA1 at both 8 h (*p* = 0.049) and 7 days (*p* = 0.042) after injury (Figure 11D). This altered trend was not observed between injury groups in the lateral or medial perforant pathways of the dentate (Figure 11D). The leftward shift of the curve suggests that CA1 pathways may generate compensatory responses to fiber volleys following TBI as previously reported following head rotational TBI in the coronal plane (Wolf et al., 2017).

## Discussion

Despite the high incidence and societal burden of TBI, the immediate injury sequelae caused by external forces and large brain tissue deformations remain poorly understood. In addition, it remains unclear the extent to which regional susceptibility affects the distribution of pathology within the brain following closed-head TBI. Here we report rapid and widespread plasma membrane permeability in cortical and hippocampal neurons acutely following closed-head rotational TBI in a large, gyrencephalic animal model. While closed-head TBI has traditionally been thought of as a predominantly white matter (i.e., axonal) injury, the current study suggests that additional injury, specifically plasmalemmal permeabilization, occurs to neuronal somata and dendrites within the cortical gray matter and hippocampus as a function of angular velocity levels. Our findings support a stereotypical order of cortical layer damage that occurs with increasing injury severity, with cortical layers II/III being most susceptible to permeability events, cortical layer V being moderately susceptible, and cortical layer VI being susceptible only in the most severe injuries. Within the white matter, permeability of neuronal somata was infrequent and axonal tract permeability was absent except in the most severe injuries. In addition to acute permeability, we observed rapid pathophysiological changes in NeuN immunoreactivity as well as increased DRP1 and CCP labeling following both single and repetitive injuries. Additionally, somato-dendritic permeability was observed within several hippocampal regions, including the hilus, dentate gyrus, CA1, and CA3, as well as in the entorhinal cortex. Furthermore, our findings support a mechanism by which acute structural injury may contribute to neurophysiological changes. Presynaptic FV slope was suppressed at 8-h and resolved by 7-days post-injury in CA1, whereas a non-significant trend in FV slope suppression was observed in the lPP. At both 8-h and 7-days, FV input lead to greater EPSP output in CA1, which suggests a compensatory response to FV suppression after TBI. Overall, our findings suggest that rapid head rotation–the loading mechanism most associated with traumatic concussion and prolonged loss-of-consciousness–can induce regionally selective neuronal permeabilization that varies by angular velocity and rotational plane of injury. Thus, neuronal mechanoporation may be a fundamental event in the pathophysiological sequelae of closed-head rotational TBI. Moving forward, direct coupling of physical and physiological responses should be carefully considered in the advancement of mechanistically-based therapeutics to attenuate secondary injury cascades and/or neurodegenerative pathways.

This exploratory study spanned a wide spectrum of injury levels emulating concussion to severe TBI in humans based on established kinematic scaling parameters (Cullen et al., 2016). Amongst the different pathologies observed, permeability and co-pathologies scaled with the biomechanics of injury. Across animals, we saw a gradient of LY permeability in the cortex and hippocampus that varied by injury severity and plane. These data suggest that a broad spectrum of neuropathology could be observed depending on the biomechanical features of the injury, which recapitulates the clinically heterogeneous and diffuse nature of human TBI. Importantly, our findings suggest a profound gray matter vulnerability that heretofore has been unappreciated in inertial TBI. In particular, we observed significant permeability of somata and neurites across the spectrum of biomechanical loading conditions. Of note, we also observed permeabilized neurons following rotational levels previously established to cause a “mild” injury phenotype (e.g., coronal plane rotation), manifesting as discrete, dense clusters of lightly-permeabilized neurons. We postulate that the extent of LY influx was proportional to the magnitude and/or duration of plasmalemmal breach upon loading. Therefore, the lighter permeability was likely indicative of reduced tissue-level excursion and strain (and hence, stress) fields. It is unlikely that all permeabilized cells are destined for cellular death, particularly following “mild” loading conditions (Geddes et al., 2003; Singleton and Povlishock, 2004; Cullen et al., 2011b; Hernandez et al., 2019), and cells surviving transient permeabilization may contribute to subsequent subacute and persistent neurophysiological pathology.

Biomechanical loading may also vary across medial versus lateral cortical structures, for instance affecting tissue level strain fields based on relative levels of angular velocities/accelerations and/or the plane of head rotation. In this study, LY permeability was frequently encountered throughout both the medial and lateral cerebral cortices, often exhibiting a “skip lesion” phenomenon of discontinuous foci of cortical permeability. The medial cortical structures with the most prominent permeability included the cingulate, visual, and motor structures. Within the lateral cortices, permeability was most frequently observed within insular, prepiriform, piriform, and parahippocampal structures. Our analysis revealed that permeability generally increased with angular velocity/acceleration in both coronal and sagittal planes. However, high angular velocity injuries in the sagittal plane generated more permeability across cortical structures relative to high angular velocity coronal plane injuries. Animals injured in the sagittal plane had unequal proportions of permeabilized cells between medial and lateral cortices. These data suggest that biomechanical loading may be unevenly distributed across cortical structures during TBI, and that cortical regions susceptible to damage may be considerately dependent on the angular velocity/acceleration and the angle of the injury sustained.

Our observations revealed that neuronal permeability preferentially occurred within particular layers of the cortex and cortical layer involvement advanced with injury severity. For the low angular velocity coronal injuries, LY permeability tended to occur in layer II and III, although certain rare instances showed isolated permeability in layer V. Higher angular velocity coronal injuries had a wider array of LY^+^ cells in layers II, III, and V. In contrast, both low and high angular velocity sagittal injuries had LY permeabilized cells throughout layers II, III, and V. LY^+^ cells were only present in layer VI after high angular velocity sagittal injuries. LY permeability was rarely seen in layers I or IV regardless of injury severity or plane. These results are in contrast to those found in a rat weight drop model where permeabilized neurons were predominantly found in layers IV and V (Farkas et al., 2006), but in line with a rat controlled cortical impact model showing permeability in superficial layers (LaPlaca et al., 2019). Differences in injury models, as well as brain architecture between rats and pigs, likely contribute to the variations in distribution of cortical permeability after injury. A combination of cell size, arborization complexity, local stresses (e.g., based on cell orientation, mechanical properties, material transitions, etc.), and/or intrinsic vulnerabilities may be contributing factors to the selective distribution of permeabilized neurons within cortical layers. Similarly, resiliency of cells in layers I and IV may be due to changes in cell density, cell size and shape, and/or the role of glial cells and horizontal fibers of passage in altering local stress formations.

Prior work from our lab has demonstrated that repetitive, moderate angular velocity, sagittal injuries separated by 15 min or 3 days induced greater neuronal permeability in subcortical structures related to control pigs (Keating et al., 2020). Of note, in that study permeability was not enhanced relative to single injuries in oculomotor regions with injuries separated by 7 days. In contrast, in the current study we observed that repetitive injuries separated by 7 days, but not 15 min, in either the coronal or sagittal plane resulted in more permeabilized neurons in the cerebral cortex relative to animals receiving a single injury. Additionally, all animals who received a second injury separated by 7 days, regardless of injury plane, demonstrated increased neuronal soma and dendritic permeability and beading within the hippocampus. Although the number of animals analyzed in the repetitive cohort of this study was limited, these data support that cortical and subcortical regions may have differing temporal intervals of vulnerability to repeat injury. Using the same model of head rotational acceleration injury, Friess et al. (2009) demonstrated that piglets subjected to repetitive injuries separated by 7 days in the axial plane had foci of enhanced axonal injury, which correlated with a worsening cognitive composite score. Additionally, they observed that repetitive injuries sustained within 1 week or less produced poorer neuropathological and neurobehavioral functional outcomes relative to animals receiving a single injury (Friess et al., 2009). Our findings and those of others suggest that cortical and hippocampal mechanoporation may contribute to the worsening neurocognitive outcomes observed with repetitive injuries in adult, and potentially pediatric, TBI.

This work expands on previous studies using rodent models of TBI that have shown immediate and sub-acute alterations in membrane permeability after injury. Studies utilizing propidium iodide have demonstrated that progressive membrane poration occurs from 5 min to 1 h post-injury after controlled cortical impact (Whalen et al., 2008). Evolving plasmalemmal changes were also demonstrated in studies that delivered dextrans before and after injury (Farkas et al., 2006). Here, neuronal uptake of pre-injury dextran was observed with and without uptake of post-injury dextran, suggesting that some membranes resealed after injury whereas other neurons had enduring plasmalemmal poration. Uptake of only post-injury dextran occurred within a subset of neurons, which further supports that secondary events after injury may contribute to membrane poration (Farkas et al., 2006). Secondary pathways involving calcium dysregulation may result in biochemical breakdown of the plasma membrane via activation of phospholipases, release of free fatty acids, increased production of reactive oxygen species (ROS), and cytoskeletal proteolysis of spectrin, tubulin, microtubule associated proteins, neurofilaments, and other membrane proteins (McIntosh et al., 1998). In this study, we acutely euthanized animals after injury in an attempt to isolate mechanically-induced membrane perturbation occurring at or immediately after injury; however, given the potential rapidity of phospholipase and/or protease activation, we cannot rule out some contribution from secondary injury cascades. However, cells with persisting/ongoing poration were unlikely to retain LY during saline washout prior to fixation. Therefore, we minimized the possibility of dye infiltration from secondary cell membrane break down due to enzymatic activation, cell death, or other mechanisms.

In addition to examining the distribution of LY permeability across different anatomical regions and cortical layers, we examined LY permeability at the cellular level. LY tended to occur in cell bodies primarily and neurites secondarily. We also observed an apparent LY uptake gradient within the soma with increasing intensity between the cytoplasm, nucleus, and nucleolus, respectively. It is unknown if this represents an increase in concentration in each of these subcellular domains. The subcellular distribution of LY mirrors previous work with HRP injected before injury in rats, which revealed peroxidase reaching the nucleus and nucleolus within 24 h of injury (Povlishock et al., 1979). Similarly, dextran uptake in the nucleus of “severely” injured neurons indicated cells that were undergoing necrosis (Farkas et al., 2006). In our work, it is notable that LY infiltrates the nucleus and nucleolus within 15 min of injury. It is unknown whether the infiltration occurs at the moment of mechanical loading and injury or whether the nucleus/nucleolus is permeable to LY that has entered the cytoplasm.

Mechanoporation may be a sentinel event amongst multiple co-occurring and evolving pathologies. Loss of NeuN immunoreactivity, a measure of neuronal injury (McPhail et al., 2004; Ünal-Çevik et al., 2004; Collombet et al., 2006; Darlot et al., 2017), has been observed chronically after rat fluid percussion (Hernandez et al., 2019) and occurs within the permeabilized neurons of porcine subcortical nuclei acutely after closed-head rotational injury (Keating et al., 2020). We demonstrate that permeabilized neurons of the cortex also have variable loss of NeuN immunoreactivity. Loss of NeuN antigenicity may indicate metabolic perturbation (Ünal-Çevik et al., 2004) or a shift in gene expression toward an immature neuronal phenotype, potentially resulting in altered neuroplasticity and reduction of dendritic branching (Dachet et al., 2014; Darlot et al., 2017). Whether the loss of NeuN antigenicity suggests a programmed–perhaps protective–stress response, an early pathophysiological response, and/or simply a change in confirmation of the antigen is unknown but warrants further examination. Furthermore, loss of NeuN was most evident in animals experiencing a single high velocity sagittal plane TBI or repetitive coronal plane TBIs separated by 7 days. Future studies should investigate features associated with these injury parameters that affect NeuN immunoreactivity following TBI.

Disruption of the plasmalemma likely causes metabolic stress through alterations of ion concentrations. Reconstitution of ionic homeostasis, in addition to cellular repair functions, likely places increased energy demands on mitochondria which may induce mitochondrial fission. Fission increases the number of mitochondria and also provides a mechanism of removing damaged mitochondria. However, during times of high cellular stress, fission can also facilitate apoptosis (Youle and van der Bliek, 2012). We demonstrated increased DRP1 (a marker of mitochondrial fission) labeling throughout the cortex, including the majority of LY^+^ cells, that was exacerbated with repetitive injuries. However, it is important to note that DRP1 positivity was not exclusively associated with neurons exhibiting membrane permeability, suggesting that mitochondrial fission can be triggered by injury mechanism(s) independent from plasmalemmal disruptions. Regardless, whether mitochondrial fission is adaptive or pathological remains unclear. Interestingly, inhibiting DRP1 after ischemic stroke prevented mitochondrial fission and the loss of mitochondrial membrane potential, thus preserving cellular energy production and resulting in a neuroprotective effect (Grohm et al., 2012). In TBI, mitochondrial stress and/or structural damage may result in release of intra-mitochondrial calcium stores into the cytoplasm. Elevations in cytoplasmic calcium concentration may aggravate ion imbalances, ultimately resulting in calcium-mediated cytoskeletal breakdown, activation of caspases, and/or delayed cell death. These secondary events likely contribute to the delayed membrane poration observed in prior studies (Farkas et al., 2006; Whalen et al., 2008; Lafrenaye et al., 2012). Alternatively, other acute intracellular events, such as glutamate imbalance, excessive production of ROS, or depletion of antioxidants and free radical scavengers may induce mitochondrial fission. Mitochondrial-targeted therapeutics may mitigate secondary injury (Cheng et al., 2012; Hiebert et al., 2015) and warrant future investigation.

Given our findings that permeabilized cells demonstrate acute loss of NeuN antigenicity and increased DRP1 immunoreactivity, it is likely that mechanoporation can induce delayed cell death. Caspase-3 is a late component of the apoptotic pathway and thought to be the “point of no return” step in programmed cell death (Clark et al., 2000). We utilized CCP, an antibody that aligns well with activated caspase-3 (Zhang et al., 2002; Chen et al., 2005), to assess the relationship between permeabilization and apoptotic pathway activation. We observed modest increases in CCP labeling after a single injury and markedly increased labeling after repetitive injury regardless of plane of injury. CCP frequently, but not exclusively, co-localized with permeabilized cells. Similarly, rodent studies have demonstrated that permeability markers (HRP, propidium iodide, dextrans) lack a one-to-one correspondence with apoptotic markers (FluoroJade, TUNEL) (Singleton and Povlishock, 2004; Whalen et al., 2008; Lafrenaye et al., 2012). In these studies, analysis of nuclear morphology demonstrated various stages of nuclear condensation indicative of either necrosis or apoptosis within permeabilized cells (Whalen et al., 2008), which suggests that a portion of permeabilized cells may die without undergoing apoptosis. Our results support that caspase activation is an early event in TBI. Likewise, caspase-related upregulation has been shown within 15–30 min of TBI and stroke (Pike et al., 1998; Chen et al., 2005). Despite rapid onset of permeability and caspase activation, TUNEL staining has not been observed within 1 h of injury (Whalen et al., 2008). Additionally, activation of caspase-3 may result in the breakdown of cytoskeletal proteins such as spectrin (Pike et al., 1998), and calpain-mediated spectrin proteolysis has been demonstrated to occur within 4 h of injury, including within permeabilized cells (Farkas et al., 2006). While calpains and caspases have been implicated in sustaining increased membrane permeability (Liu and Schnellmann, 2003), other studies have shown that calpains may play a role in resealing the membrane (Howard et al., 1999). Further studies are needed to elucidate the fate of cells exhibiting acute permeabilization (e.g., survival, apoptosis, necrosis), especially in the context of repetitive injuries, where cell death thresholds may be reduced.

Hippocampal-dependent deficits, including learning and memory formation, are a prominent feature of TBI. Previous studies of porcine closed-head rotational injury have demonstrated functional alterations within the hippocampus despite a lack of corresponding axonal pathology (Wolf et al., 2017). Our studies did not reveal axonal permeability within the hippocampus, which further underscore the potential resiliency of axons to plasmalemmal disruptions in inertial/rotational TBI. However, LY^+^ soma and dendritic beading were prevalent throughout the hippocampal circuitry (e.g., dentate gyrus, hilus, CA1, CA3, entorhinal cortex) following even a single rotational injury. Furthermore, dentate granule cells often displayed either permeabilized soma with a “string-of-pearls” LY distribution in their dendrites, or a more uniform LY distribution throughout the somato-dendritic compartment. Given the rapid onset of neuronal membrane permeability and dendritic beading, these are likely acute biophysical disruptions due to diffuse strain fields.

Rapid damage of dendrites may have profound functional implications for hippocampal circuitry, specifically for dentate granule cells that receive inputs from the entorhinal cortex and thus serve as the input stage of hippocampal circuitry. To investigate the idea that TBI-induced somato-dendritic damage may contribute to early neurophysiological dysfunction, we performed electrophysiological studies of acute hippocampal slices from a cohort of animals undergoing high angular velocity head rotation in the sagittal plane. Interestingly, we saw that presynaptic FV slope was suppressed 8-h after injury in CA1 and trending in the lPP, but was resolved by 7-days post-injury. This may suggest a channel level response as has previously been reported with *in vitro* models and supported by findings following coronal plane head rotation in this model (Wolf et al., 2001, 2017). In contrast with the coronal plane, these changes had resolved at the 7-day time point, suggesting that plane of rotation may influence the length of time that this effect is present post-injury in the hippocampus. In addition, a given FV input led to a greater EPSP output at both 8-h and 7-days after injury, suggesting that compensatory responses to FVs may be generated after TBI. Follow-up studies are necessary to elucidate structural changes at the level of dendritic arborization, spines, and/or synapses that may contribute to these functional changes, as well as further identifying the mechanisms of the reduction in fiber volleys and enhancement of dendritic response, potentially via homeostatic processes.

Overall, our findings provide further evidence for “multiple cell injury phenotypes” in acute TBI (Singleton and Povlishock, 2004; Stone et al., 2004; Johnson et al., 2016). Regional variability in biomechanical susceptibility likely contributes to selective sensitivity to cytoskeletal disruptions, a prominent feature of DAI, as well as to mechanoporation. Interestingly, we found that white matter tract permeability was relatively rare with only the highest angular velocity head rotation in the sagittal plane inducing appreciable primary axonal permeability. This finding is partially supported by seminal studies done in the 1990’s using a feline model of fluid percussion injury that showed that following mild TBI, axons did not exhibit flooding with HRP yet displayed characteristic neurofilament compaction and axonal swelling consistent with classic DAI neuropathology (Pettus et al., 1994; Pettus and Povlishock, 1996; Okonkwo et al., 1998). However, these studies did show focal axolemmal permeability to HRP following moderate-to-severe TBI that was also associated with local mitochondrial abnormalities in addition to increased neurofilament packing and microtubule loss. Together, these findings may suggest that the open cranium and more focal nature of fluid percussion injury may generate larger localized (e.g., white matter) stress-strain fields than those generated by closed-head rotational TBI, such as the porcine model used in the current study. Collectively, our current findings coupled with prior studies suggest that the most sensitive (i.e., occurring with lower biomechanical thresholds), principal physical response to axonal loading involves cytoskeletal, not plasmalemmal, damage (Chen et al., 1999; Smith et al., 1999b; Tang-Schomer et al., 2010, 2012). This may be due to the anisotropy of white matter tracts reducing the complexity of axonal-level strain fields following diffuse loading (Cullen and LaPlaca, 2006). Axonal cytoskeletal damage likely directly contributes to transport interruptions and disconnection thereby causing axons to degenerate with functional consequences commensurate with the type and extent of axonal loss. Our findings also suggest that neuronal mechanoporation contributes to neurophysiological outcomes after rotational TBI. Following plasmalemmal damage, neurons may be chronically dysfunctional–rather than permanently lost–and therefore may play a role in circuit-level deficits. In addition, at least a portion of degenerating axons in subcortical white matter may be a secondary consequence of primary injury to long-projecting neurons residing in cortical layers V or VI, which would be consistent with DAI pathology generally peaking over hours to days post-injury (Ross et al., 1994; Chen et al., 1999; Browne et al., 2011; Johnson et al., 2018).

The cortical patterns of permeability we observed acutely following injury are consistent with the distribution of pathology frequently encountered in human TBI. Clinically, multi-focal lesions of DAI and associated edema are frequently observed at gray-white matter junctions. Our data suggests additional loading-induced neuronal somatic injury at gray-white matter interfaces that may be a component of the lesions and edema observed in patients after TBI. Additionally, our findings in animals receiving multiple injuries suggests that repetitive injuries may enhance susceptibility to neuronal permeability within both the cortex and hippocampus. In addition, the distribution of permeability observed in this study is consistent with the regions of decreased cortical thickness observed in magnetic resonance imaging studies of young to middle-aged adults with multiple mild TBIs (List et al., 2015). However, it remains unclear how multiple injuries, as well as the timing between injuries, effects injury thresholds, distributions, mechanisms, and pathophysiological responses. Furthermore, the relationship between permeabilized cells and neuro-inflammation is poorly characterized, but may reveal therapeutic targets to decrease evolving damage and chronic deleterious sequelae after injury. Additionally, as this study exclusively investigated the relationships between rotational plane, injury kinematics, brain region, and pathological distribution in female swine, future studies should investigate if there is an effect of biological sex on the extent, susceptibility, or distribution of plasmalemmal pathology. Future studies should also more thoroughly examine the incidence of neuronal, dendritic, and axonal permeability in context with other mechanisms of pathological mechanosensation (e.g., cytoskeletal discontinuities) and classically-described acute pathologies (e.g., DAI, neuronal death) based on kinematic parameters (e.g., angular velocity and acceleration), plane of loading, and predicted tissue- and cell-level strain fields in order to create a comprehensive catalog of the thresholds, relevance, and consequences of biophysical disruptions in closed-head TBI.

## Conclusion

This report demonstrates the preferential vulnerability of neuronal somata and dendrites to acute alterations in plasmalemmal permeability using a porcine model that closely mimics the biomechanics of closed-head diffuse TBI in humans. This pathology was found across various anatomical levels of the cerebral cortex and hippocampus, with the distribution of permeabilized neurons typically presenting in multi-focal patches within gyri spanned by seemingly unaffected regions. When present, these patches of permeabilized neurons were generally found in layers II/III of the cortex, with the additional involvement of layers V and VI with escalating biomechanical loading. Axolemmal permeability in sub-cortical white matter tracts was rarely observed, supporting previous conclusions that head rotational injury primarily leads to both axonal channel dysfunction and cytoskeletal discontinuities rather than axolemmal disruptions. Our findings suggest the presence of significant dendritic and somatic permeability that has been unappreciated in head rotational injury but may influence subsequent pathophysiological progression and neurophysiological deficits. In the hippocampus, neuronal somatic permeability and dendritic beading occurred in parallel with neurophysiological disruptions of the relationship between presynaptic and postsynaptic processes. Examination of co-pathologies that occurred with membrane permeability showed that traumatized neurons were undergoing a myriad of responses including altered NeuN immunoreactivity, increased mitochondrial fission, and caspase pathway activation. This work implicates the mechanical loading of cells during TBI as an important trigger causing escalating pathophysiological cascades, with these sequelae perhaps occurring in a more rapid time frame than has been previously appreciated. An improved understanding of the biomechanical etiology and resulting pathology of closed-head rotational TBI is necessary to accurately determine injury tolerances, validate the efficacy of safety standards and protective equipment, as well as to develop mechanistically-based therapeutics to mitigate the resulting neurophysiological dysfunction and neurodegeneration.

## Data availability statement

The raw data supporting the conclusions of this article will be made available by the authors, without undue reservation.

## Ethics statement

The animal study was reviewed and approved by the Institutional Animal Care and Use Committee, University of Pennsylvania.

## Author contributions

DC and KB conceived of and designed the experiments. JH, CM, KB, KW, CK, DB, BJ, JW, and DC performed material preparation, data collection, and analysis. JH and DC wrote the first draft of the manuscript. All authors provided critical feedback and approved the final manuscript.
